# Factors associated with mortality in mechanically ventilated patients with severe acute respiratory syndrome due to COVID-19 evolution

**DOI:** 10.5935/2965-2774.20230203-en

**Published:** 2023

**Authors:** João Paulo Arruda de Oliveira, Andreia Cristina Travassos Costa, Agnaldo José Lopes, Arthur de Sá Ferreira, Luis Felipe da Fonseca Reis

**Affiliations:** 1 Postgraduate Program in Rehabilitation Sciences, Centro Universitário Augusto Motta - Rio de Janeiro (RJ), Brazil; 2 Intensive Care Unit, Hospital Oswaldo Cruz - Tocantins (TO), Brazil

**Keywords:** Respiratory distress syndrome, COVID-19, Coronavirus infections, SARS-CoV-2, Respiration, artificial, Respiratory mechanics, Mortality

## Abstract

**Objectives:**

To evaluate the factors associated with mortality in mechanically ventilated patients with acute respiratory distress syndrome due to COVID-19.

**Methods:**

This was a retrospective, multicenter cohort study that included 425 mechanically ventilated adult patients with COVID-19 admitted to 4 intensive care units. Clinical data comprising the SOFA score, laboratory data and mechanical characteristics of the respiratory system were collected in a standardized way immediately after the start of invasive mechanical ventilation. The risk factors for death were analyzed using Cox regression to estimate the risk ratios and their respective 95%CIs.

**Results:**

Body mass index (RR 1.17; 95%CI 1.11 - 1.20; p < 0.001), SOFA score (RR 1.39; 95%CI 1.31 - 1.49; p < 0.001) and driving pressure (RR 1.24; 95%CI 1.21 - 1.29; p < 0.001) were considered independent factors associated with mortality in mechanically ventilated patients with acute respiratory distress syndrome due to COVID-19. Respiratory system compliance (RR 0.92; 95%CI 0.90 - 0.93; p < 0.001) was associated with lower mortality. The comparative analysis of the survival curves indicated that patients with respiratory system compliance (< 30mL/cmH_2_O), a higher SOFA score (> 5 points) and higher driving pressure (> 14cmH_2_O) were more significantly associated with the outcome of death at 28 days and 60 days.

**Conclusion:**

Patients with a body mass index > 32kg/m^2^, respiratory system compliance < 30mL/cmH_2_O, driving pressure > 14cmH_2_O and SOFA score > 5.8 immediately after the initiation of invasive ventilatory support had worse outcomes, and independent risk factors were associated with higher mortality in this population.

## INTRODUCTION

Acute respiratory distress syndrome (ARDS) due to the evolution of coronavirus 2019 (COVID-19) is characterized by severe acute lung injury with alteration of the permeability of the pulmonary capillaries and an aberrant inflammatory response of the host, with rapidly evolving refractory hypoxemia, associated or not with disseminated intravascular coagulation, which causes high mortality rates, especially in 2020.^(1)^ Brazil was considered an epicenter of the disease in 2021, surpassed only by the United States.^(2)^ Hospital mortality was high, even among patients younger than 60 years, and reached 80% among patients who were mechanically ventilated.^(3)^

Males aged > 60 years and with comorbidities are more likely to die in the intensive care unit (ICU).^(4)^ Among hospitalized patients, 40% develop ARDS, requiring invasive mechanical ventilation (MV).^(5)^ Acute respiratory distress syndrome due to COVID-19 has a complex pathophysiology that involves variations in the degrees of pulmonary infiltration, thrombotic injury and heterogeneous respiratory mechanics.^(6)^

Studies suggest that protective MV is established through the use of lower tidal volumes (Vt) up to 6mL/kg of predicted weight, distension pressures or driving pressure < 15cmH_2_O (ideally < 13cmH_2_O) and a plateau pressure < 30cmH_2_O.^(7,8,9,10,11)^ Due to the heterogeneity of ARDS, ventilatory strategies must be individualized to obtain better outcomes and, consequently, minimize the risk of ventilator-induced lung injury (VILI).^(1,12,13,14)^

Predictors of worse outcomes collected at patient admission may provide useful information to support clinical and public health decisions regarding invasive M V.

The aim of this study was to evaluate the factors associated with mortality in mechanically ventilated patients with ARDS due to COVID-19 evolution.

## METHODS

This was an observational, longitudinal, retrospective, multicenter cohort study conducted in 4 adult ICUs in two Brazilian states. Patients aged ≥ 18 years, under M V, diagnosed with ARDS of pulmonary etiology secondary to COVID-19 infection, and met the following Berlin criteria were included in the study:^(15)^ time of exposure to the risk factor < 7 days, presence of bilateral pulmonary infiltrates of noncardiac origin (absence of signs of left atrial hypertension), confirmed diagnosis through computed tomography (CT) of the chest, refractory hypoxemia, and partial pressure oxygen/fraction of inspired oxygen (PaO_2_/FiO_2_) < 300 and minimum positive pressure of 5cmH_2_O after initial titration of positive end-expiratory pressure (PEEP) and adjustment of minimum FiO_2_ to maintain arterial saturation between 92 and 96% and PaO_2_ > 65mmHg.

Patients admitted from other hospitals not participating in the study, patients who progressed to orotracheal intubation (OTI) in wards or hospitalization units; patients who were intubated for other causes, even if they later progressed to SARS-CoV-2 coinfection; patients without clinical criteria for ARDS; and patients with incomplete data related to ventilatory parameters and/or baseline clinical data were excluded.

The study protocol was approved by the institutional ethics committee, and the requirement for obtaining informed consent forms was waived (CAAE: 53152221.3.0000.5235), respecting all ethical principles and reported in accordance with the Strengthening the Reporting of Observational Studies in Epidemiology (STROBE).

This is a nonprobabilistic convenience sample. All data were collected using a research protocol prepared by the researchers. Data recorded immediately after OTI and clinical stabilization after OTI were considered. Anthropometric data (weight, height, and body mass index - BMI, kg/m^2^) were obtained from the admission records. Patients with a BMI ≥ 30kg/m^2^ were considered obese. mechanical ventilation variables, such as ventilatory mode, Vt, inspiratory time, inspiratory flow, ideal PEEP (after decremental titration performed according to institutional protocols), fraction of inspired oxygen, peak pressure, plateau pressure and mean airway pressure; ventilatory mechanics data (static compliance and airway resistance); arterial blood gas analysis results; and data from the derivative measures of oxygenation, such as PaO_2_/FiO_2_, alveolar-arterial oxygen difference (D(A-a)O_2_), and arterial oxygen content (CaO_2_) were obtained from the data collected from the first blood gas analysis after OTI and invasive M V, after PEEP titration and after adjustment of the minimum FiO2 to maintain PaO_2_ > 65mmHg and oxygen saturation (SaO_2_) 92 - 96%. Laboratory test results (red blood cell count, hemoglobin, hematocrit, lactate, creatinine, total platelets and bilirubin); information on weight and height for the calculation of BMI; levels of agitation and sedation, as measured using the Richmond scale (RASS - Richmond Agitation-Sedation Scale); neurological assessment results, as determined using the Glasgow Coma Scale; hemodynamic function (mean arterial pressure and use of vasoactive drugs); data on the use of neuromuscular blockers and sedoanalgesia; and clinical severity scores, as determined using the Sequential Organ Failure Assessment (SOFA) were obtained from the electronic medical records of each participant. Patients were followed up from admission to ICU discharge or death. All patients with a PaO_2_/FiO_2_ < 150 were administered neuromuscular blocking agents and were ventilated in the prone position for at least 16 hours from the first 48 hours of evolution, followed by prone MV while the patients were responding to and requiring the intervention.

### Statistical analysis

Continuous variables are expressed as the mean ± standard deviation (SD) and 95% confidence interval (95%CI). The groups were compared using one-way analysis of variance (ANOVA), as appropriate, based on the Shapiro‒Wilk normality test. Categorical variables are expressed as percentages (%) and were compared using the chi-square test. The patients were divided into three analysis cohorts based on an *a priori* hypothesis, based exclusively on mechanical criteria, an approach supported by previous studies by Robba et al.^(12)^ and Gattinoni et al.^(13)^ Thus, the patients were divided by mechanical characteristics measured immediately after OTI into three analysis cohorts: low compliance (LC), i.e., respiratory system compliance (Crs) < 30mL/cmH_2_O; intermediate compliance (IC), i.e., 30mL/cmH_2_O < Crs < 45 mL/cmH_2_O; and high compliance (HC), i.e., Crs > 45mL/cmH_2_O. The incidence of the outcome was calculated for each group, and the follow-up time in the denominator of the incidence rate was 28 days and 60 days after OTI.

The survival analysis was performed using Kaplan-Meier estimators, and for comparative analyses, the log-rank test was used. The risk factors for death were analyzed using Cox regression to estimate the risk ratios (RRs) and their respective 95% CIs to establish the predictors related to mortality in mechanically ventilated patients with ARDS.

To assess whether there were one or more prognostic factors, multivariate logistic regression was performed to determine the risk of the outcome (beta exponential); which control variables were associated with the outcome (mortality and time to outcome); and whether there was a cutoff point and, from there, if the risk increased or decreased. Logistic regression aids in modelling the occurrence (or nonoccurrence) of an event (zero or one) of a binary variable and its relationship with continuous variables.

All statistical analyses were conducted by an independent statistician who did not participate in any of the stages of the project and was not part of the research group that conceived of or conducted this study. Statistical analysis was performed using Jamovi® (https://www.jamovi.org/), and p < 0.05 was considered statistically significant.

## RESULTS

**Figure 1:**
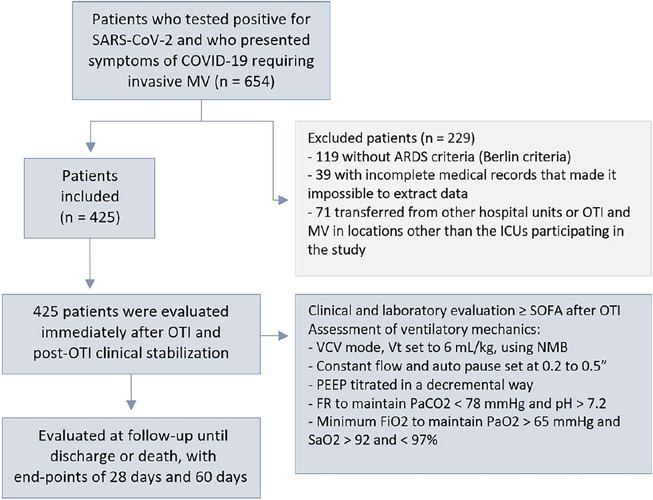
Study flowchart

Between March 2020 and June 2021, 654 patients were admitted to the participating ICUs due to the clinical evolution of COVID-19. Of these, the data of 425 individuals admitted to the participating ICUs who progressed to OTI and invasive MV due to the evolution of SARS-CoV-2 infection were retrospectively analyzed (Figure 1). Among the patients included in this cohort, most were hospitalized in 2020 (221/425, 52%), with 48% (204/425) of hospitalizations occurring in 2021. Most participants were male (n = 291; 68.5%). The participants had a mean length of hospital stay of 20.88 days (95%CI 19.40 - 22.36), a mean age of 61.59 years (95%CI 60.33 - 62.85), and a mean BMI of 28.49kg/m^2^ (95%CI 27.84 - 29.15).

SARS-CoV-2 - severe acute respiratory syndrome coronavirus 2; MV - mechanical ventilation; ARDS - acute respiratory distress syndrome; OTI - orotracheal intubation; ICU - intensive care unit; SOFA - Sequential Organ Failure Assessment; VCV - volume controlled ventilation; Vt - tidal volume; NMB - neuromuscular blocking agent; PEEP - positive end-expiratory pressure; RR - respiratory rate; PaCO_2_ - partial pressure of carbon dioxide; FiO_2_ - fraction of inspired oxygen; PaO_2_ - partial pressure of oxygen; SaO_2_ - oxygen saturation.

The mean SOFA score was 5.82 points (95%CI 5.65 - 6.00). The average duration on MV was 18.07 days (95%CI 16.79 - 19.34). Data related to the ventilatory variables adjusted immediately after OTI were analyzed; Vt, on average, was 6.56mL/kg of predicted body weight (95%CI 6.42 - 6.71), and the mean PEEP was 11.11cm/H_2_O (95%CI 10.88 - 11.33). The mean driving pressure was 15.24cm/H_2_O (95%CI 14.91 - 15.58), and the mean Crs was 30.38mL/cmH_2_O (95%CI 29.51 - 31.25). The general characteristics of the sample are provided in table 1.

**Table 1:** General characteristics of the total sample of mechanically ventilated patients with COVID-19

Clinical features	n/n total (%)	Mean ± SD	IC95%
2020	221/425 (52)		
2021	204/425 (48)		
Sex			
Male	291/425 (68.5)		
Female	134/425 (31.5)		
Overall mortality	425/254 (59.8)		
Age (years)		61.59 ± 13.24	60.33 - 62.85
BMI (kg/m^2^)		28.49 ± 6.86	27.84 - 29.15
SOFA score		5.82 ± 1.85	5.65 - 6.00
Days of hospitalization		20.88 ± 15.55	19.40 - 22.36
MV time (days)		18.07 ± 13.40	16.79 - 19.34
Sedoanalgesia (days)		18.56 ± 13.64	17.26 - 19.85
NMB (days)		3.23 ± 2.71	2.97 - 3.48
Hemodynamics			
MAP (mmHg)		82.14 ± 21.33	80.11 - 84.17
VAD (days)		17.91 ± 13.02	16.67 - 19.15
Ventilatory support			
Vt (mL/kg)		6.56 ± 1.50	6.42 - 6.71
FiO_2_ (%)		77.44 ± 20.63	75.48 - 79.40
PEEP (cmH_2_O)		11.11 ± 2.33	10.88 - 11.33
Plateau (cmH_2_O)		26.35 ± 4.24	25.94 - 26.75
Driving pressure (cmH_2_O)		15.24 ± 3.52	14.91 - 15.58
Crs (mL/cmH_2_O)		30.38 ± 9.14	29.51 - 31.25
Laboratory tests			
pH		7.36 ± 0.14	7.35 - 7.37
PaCO_2_ (mmHg)		46.27 ± 19.53	44.41 - 48.13
Lactate (mmol)		2.02 ± 2.69	1.77 - 2.28
PaO_2_/FiO_2_		156.35 ± 80.82	148.66 - 164.03
D(A-a)O_2_ (mmHg)		430.89 ± 158.81	415.79 - 445.99
CaO_2_ (g/dL/100mL)		12.08 ± 1.97	11.90 - 12.27

SD - standard deviation; 95%CI - 95% confidence interval; BMI - body mass index; SOFA - *Sequential Organ Failure Assessment*; MV - mechanical ventilation; NMB - neuromuscular blocking agent; MAP - mean arterial pressure; VAD - vasoactive drug; Vt - tidal volume; FiO_2_ - fraction of inspired oxygen; PEEP - positive end-expiratory pressure; Plateau - plateau pressure; Crs - respiratory system compliance; PaCO_2_ - partial pressure of carbon dioxide; PaO_2_ - partial pressure of oxygen; FiO_2_ - fraction of inspired oxygen; D(A-a)O_2_ - alveolar-arterial oxygen difference; CaO_2_ - arterial oxygen content

Among the patients, 49.41% had LC, 30.35% had IC, and 20.23% had HC. When comparing the clinical characteristics among the cohorts, individuals with LC were older (LC = 63.83 years, 95%CI 62.14 - 65.51; IC = 59.56 years, 95%CI 57.08 - 62.05; HC = 59.14 years, 95%CI 56.48 - 61.79; p = 0.002) and had a significantly higher BMI (LC = 30.05kg/m^2^, 95%CI 29.09 - 31.01; IC = 27.54kg/m^2^, 95%CI 26.45 - 28.63; HC = 26.11kg/m^2^, 95%CI 24.81 - 27.41; p < 0.001). Patients in the HC group had a lower severity score (LC = 6.20, 95%CI 5.96 - 6.44; IC = 5.78, 95%CI 5.44 - 6.12; HC = 4.95, 95%CI 4.61 - 5.29; p < 0.001).

**Figure 2:**
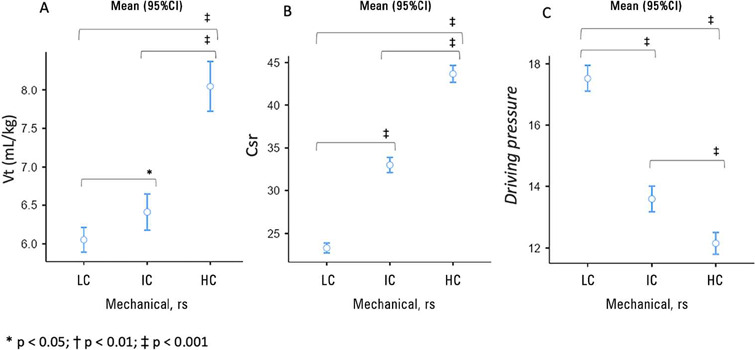
Comparisons of ventilatory variables tidal volume (A), respiratory system compliance (B) and driving pressure (C) among groups with different mechanical profiles

Regarding hemodynamic behavior, there was no significant difference among the groups regarding the use of vasopressors. Among the ventilatory and respiratory mechanic variables, the HC group had the highest mean Vt, and the patients in the LC group had the lowest Vt (LC = 6.05mL/kg, 95%CI 5.89 - 6.21; IC = 6.41mL/kg, 95%CI 6.18 - 6.64; HC = 8.05mL/kg, 95%CI 7.72 - 8.37; p < 0.001) (Figures 2A and 2B). Higher driving pressures were measured at the time of initiation of MV for individuals in the LC group, followed by IC and HC groups (LC = 17.52cmH_2_O, 95%CI 17.10 - 17.94; IC = 13.58cmH_2_O, 95%CI 13.17 - 14.00; HC = 12.15cmH_2_O, 95%CI 11.80 - 12.50; p < 0.001) (Figure 2C). The mean PEEP was lowest in the HC group (LC = 11.38cmH_2_O, 95%CI 11.02 - 11.74; IC = 11.26cmH_2_O, 95%CI 10.94 - 11.58; HC = 10.19cmH_2_O, 95%CI 9.80 - 10.59; p < 0.001). Comparisons among groups are shown in table 2.

Patients were stratified into groups of low compliance, intermediate compliance and high respiratory system compliance.

Vt - tidal volume; 95%CI - 95% confidence interval; LC - low compliance; IC - intermediate compliance; HC - high compliance; rs - respiratory system.

**Table 2:** General characteristics of the sample by clinical profile (respiratory compliance established immediately after orotracheal intubation)

Characteristics	Crs < 30mL/cmH_2_O (n = 210)		30 mL/cmH_2_O < Crs < 45mL/cmH_2_O (n = 129)		Crs > 45mL/cmH_2_O (n = 86)	
	Mean	IC95%	Mean	IC95%	Mean	IC95%
Age (years)	63.83	62.14 - 65.51*†	59.56	57.08 - 62.05	59.14	56.48 - 61.79
BMI (kg/m^2^)	30.05	29.09 - 31.01*†	27.54	26.45 - 28.63	26.11	24.81 - 27.41
SOFA	6.20	5.96 - 6.44*†	5.78	5.44 - 6.12‡	4.95	4.61 - 5.29
Days of hospitalization	15.97	14.22 - 17.71	21.38	19.06 - 23.70	32.11	28.22 - 36.00
MV time (days)	14.96	13.36 - 16.56	17.65	15.70 - 19.59	26.27	22.82 - 29.73
Sedoanalgesia (days)	15.46	13.79 - 17.12	18.51	16.55 - 20.48	26.16	22.63 - 29.69
NMB (days)	3.28	2.90 - 3.66	2.93	2.52 - 3.33	3.52	2.90 - 4.14
Hemodynamics						
MAP (mmHg)	85.40	82.24 - 88.56	81.37	77.94 - 84.71	75.39	71.89 - 78.88
Ventilatory support						
Vt (mL/kg)	6.05	5.89 - 6.21*†	6.41	6.17 -6.64‡	8.04	7.72 - 8.36
FiO_2_ (%)	80.63	77.82 - 83.45*	72.45	69.00 -75.89	77.11	72.93 - 81.30
PEEP (cmH_2_O)	11.38	11.02 - 11.74†	11.26	10.94 -11.58‡	10.19	9.80 - 10.59
Plateau (cmH_2_O)	28.90	28.40 - 29.40	24.85	24.34 - 25.36	22.34	21.78 - 22.91
Driving pressure (cmH_2_O)	17.52	17.10 - 17.94*†	13.58	13.17 - 14.00‡	12.15	11.80 - 12.50
Crs (mL/cmH_2_O)	23.31	22.73 - 23.88*†	33.02	32.15 - 33.89‡	43.67	42.70 - 44.64
Laboratory tests						
pH	7.35	7.33 - 7.38	7.34	7.32 - 7.36	7.37	7.34 - 7.40
PaCO_2_ (mmHg)	47.04	44.20 - 49.88	46.56	43.41 - 49.72	43.92	40.20 - 47.63
Lactate (mmol)	2.23	1.85 - 2.62	1.88	1.44 - 2.32	1.71	1.18 - 2.24
PaO_2_/FIO_2_	152.61	140.88 - 164.35	164.38	150.60 - 178.17	153.39	139.48 - 167.31
D(A-a)O_2_ (mmHg)	464.02	444.54 - 483.49*	380.69	351.35 - 410.02‡	425.28	392.03 - 458.52
CaO_2_ (g/dL/100 mL)	11.99	11.77-12.22	12.20	11.80 - 12.61	12.11	11.67 - 12.54
Hb (g/dL)	9.37	9.05 - 9.70	9.70	9.24 - 10.15	9.60	9.10 - 10.09

Crs - respiratory system compliance; 95%CI - 95% confidence interval; BMI - body mass index; SOFA - Sequential Organ Failure Assessment; MV - mechanical ventilation; NMB - neuromuscular blocking agent; MAP - mean arterial pressure; Vt - tidal volume; FiO_2_ - fraction of inspired oxygen; PEEP - positive end-expiratory pressure; Plateau - plateau pressure; PaCO_2_ - partial pressure of carbon dioxide; PaO_2_ - partial pressure of oxygen; D(A-a)O_2_ - alveolar-arterial oxygen difference; CaO_2_ - arterial oxygen content; Hb - hemoglobin. p < 0.05 for in the intergroup comparisons * low compliance *versus* intermediate compliance; † low compliance *versus* high compliance; ‡ intermediate compliance *versus* high compliance.

**Figure 3:**
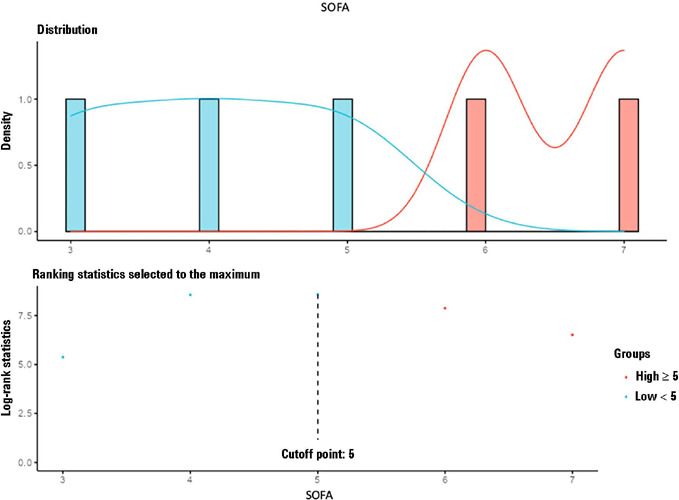
Cutoff score for the Sequential Organ Failure Assessment established by Cox models and classified by the log-rank test

**Figure 4:**
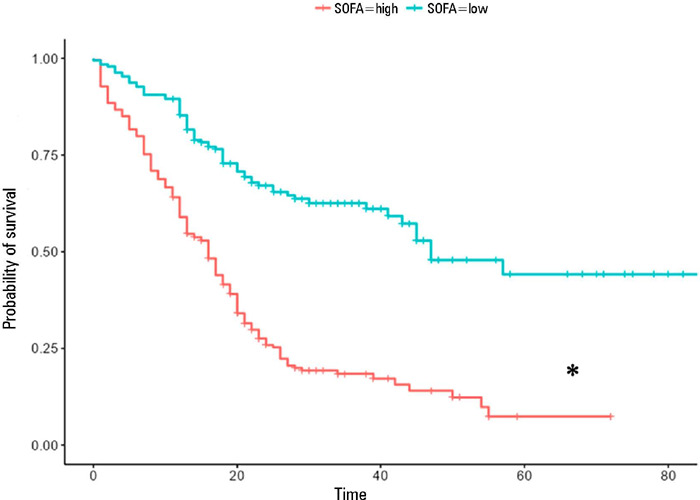
Comparative survival analysis using Kaplan-Meier estimators based on the grouping defined by the Sequential Organ Failure Assessment cutoff score established by the Cox model, i.e., < 5 points and ≥ 5 points. The cutoff score for the Sequential Organ Failure Assessment established by the model was 5 points

**Figure 5:**
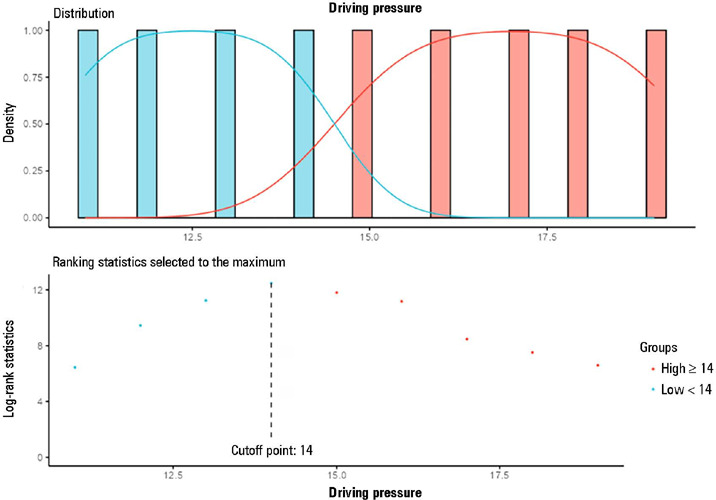
Driving pressure cutoff point established by Cox models and classified by the log-rank test

**Figure 6:**
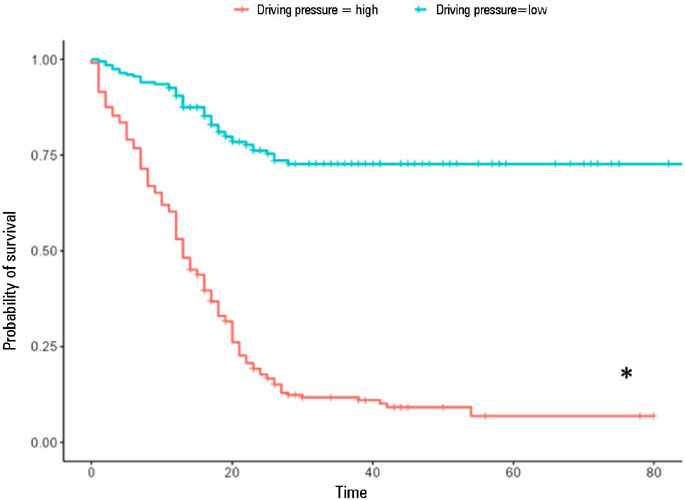
Comparative survival analysis using Kaplan-Meier estimators based on the grouping defined by the driving pressure cutoff point [(driving pressure: plateau pressure, obtained after a short pause in volume-controlled ventilation, subtracted from total positive end-expiratory pressure)] established by the Cox model, i.e., < 14mL/cmH_2_O and ≥ 14mL/cmH_2_O. The driving pressure cutoff point established by the model was 14cmH_2_O

Overall mortality was 59.8% (n = 254), with a higher prevalence in the LC group (85.2%); in the IC and HC groups, the mortality rate were 45.6% and 19.0%, respectively, throughout the study period. When analyzing the predictors of mortality, a higher BMI (≥ 30kg/m^2^) was associated with a 17% higher risk of mortality (RR 1.17; 95%CI 1.11 - 1.20; p < 0.001), and an increase of one point increase in the SOFA score above the cutoff point (Figure 3) was associated with a 39% greater chance of mortality (RR 1.39; 95%CI 1.31-1.49; p < 0.001). The mortality predictors are described in table 3. Patients with SOFA scores below 5 had a higher probability of survival (Figure 4), i.e., 85% at 12 days, 62.6% at 36 days and 44.2% at 60 days. Additionally, each 1 cmH_2_O increase in driving pressure above the cutoff point (Figure 5) was associated with 24% greater odds of death (RR 1.24; 1.21-1.29; p < 0.001]) (Table 3), in addition to reducing the probability of survival among those individuals during follow-up (Figure 6).

SOFA - Sequential Organ Failure Assessment.

* Patients with SOFA ≥ 5 points had significantly lower 28-day and 60-day survival than did patients with SOFA < 5 points.

SOFA - Sequential Organ Failure Assessment.

* Patients with a driving pressure ³ of 14 cmH_2_O had significantly lower 28-day survival and 60-day survival than did patients with a driving pressure < 14cmH_2_O.

**Table 3:** Cox regression analysis to determine the predictive factors for mortality in mechanically ventilated patients with COVID-19

Predictors	Mean ± SD	RR (Univariate)	RR (Multivariate)
BMI	28.5 ± 6.9	1.17 (1.11 - 1.20), p < 0.001)	1.17 (1.11 - 1.20), p < 0.001)
SOFA	5.8 ± 1.9	1.39 (1.30 - 1.49), p < 0.001)	1.39 (1.30 - 1.49), p < 0.001)
Driving pressure	15.2 ± 3.5	1.24 (1.2 1 - 1.29), p < 0.001)	1.24 (1.21 - 1.29), p < 0.001)
Crs	30.4 ± 9.1	0.92 (0.90 - 0.93), p < 0.001)	0.92 (0.90 - 0.93), p < 0.001)

RR - risk ratio; SD - standard deviation; BMI - body mass index; SOFA - Sequential Organ Failure Assessment; Crs - respiratory system compliance.

**Figure 7:**
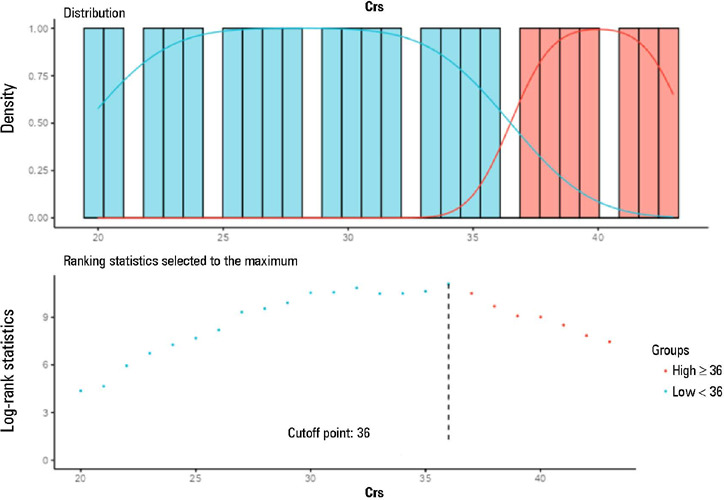
Cutoff point for respiratory system compliance established by Cox models and classified by the log-rank test

**Figure 8:**
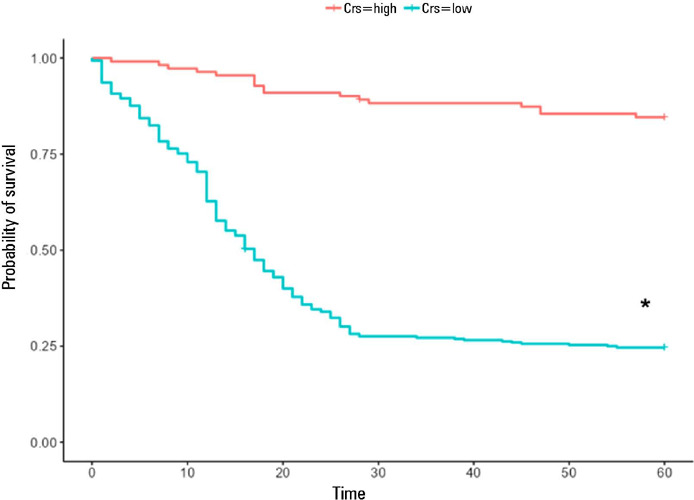
Comparative survival analysis using Kaplan-Meier estimators based on the grouping defined by the respiratory system compliance cutoff point (Crs, mL/cmH_2_O) established by the Cox model, i.e., Crs < 36mL/cmH_2_O and Crs ≥ 36mL/cmH_2_O, and low respiratory system compliance

**Figure 9:**
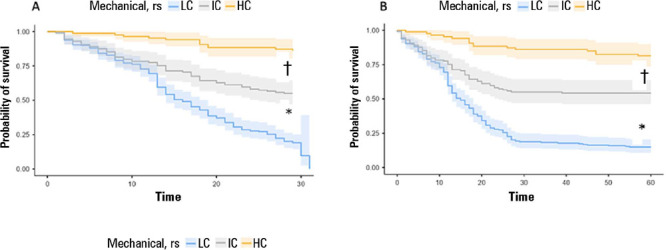
Comparative survival analysis using Kaplan-Meier estimators for patients stratified by mechanical characteristics of the respiratory system at 28 days (A) and 60 days (B). Comparisons were established using the log rank test, considering p < 0.05 as significant and with the differences between the Kaplan-Meier estimators contained within the 95% confidence interval

Patients with Csr above 36mL/cmH_2_O (Figure 7) was associated with lower mortality (Table 3) and, consequently, with a higher probability of survival (Figure 8), which was 96.4% at 12 days, 88.3% at 36 days and 84.6% at 60 days. The comparative analysis of the survival curves showed that patients with a Crs < 30mL/cmH_2_O had a higher probability of death at 28 days and 60 days than did patients with IC (p < 0.001) and HC (p < 0.001), respectively (Figures 9A and 9B).

Crs - respiratory system compliance.

Comparisons were established using the log rank test, considering p < 0.05 as significant and with the differences between the Kaplan-Meier estimators contained within the 95% confidence interval. * Patients with respiratory system compliance < 36mL/cmH_2_O had significantly lower 28-day and 60-day survival than did patients with Crs ≥ 36mL/cmH_2_O.

Crs - respiratory system compliance.

* The low compliance group had significantly lower 28-day and 60-day survival than did the IC group; † The intermediate compliance group had significantly lower 28-day and 60-day survival than did the high compliance group. rs - respiratory system; LC - low compliance; IC - intermediate compliance; HC - high compliance.

## DISCUSSION

This retrospective multicenter observational study included mechanically ventilated patients with ARDS due to COVID-19 at four Brazilian hospitals. Obesity-related factors, low Crs, higher SOFA score and driving pressure were independent prognostic factors associated with mortality at the 28- and 60-day follow-ups.

The results indicated that obesity (BMI ≥ 30kg/m^2^) was an independent predictive factor associated with mortality. Patients with obesity exhibit inflammatory cascade activation with a higher concentration of proinflammatory cytokines produced by adipose tissue, compromising the immune response in addition to being associated with hypercoagulability disorders, which are known to be associated with a worse prognosis in the evolution of COVID-19.^(16)^ Another study concluded that obesity was associated with worse COVID-19 outcomes, resulting in a higher risk of hospitalization and ICU admission, requiring the use of invasive MV, and higher chances of death.^(17)^

A SOFA score greater than 5 points was associated with higher mortality. These results reflect the degree of multiple organ dysfunction and disease severity. SOFA at admission allowed the establishment of COX models to establish to what extent this assessment predicts risk over the disease course, as already demonstrated in a study with serial assessments.^(18)^ Another study evaluated the reliability of SOFA on admission in predicting mortality in patients with ARDS due to COVID-19 and concluded that this score had robust potential for predicting mortality, with an area under the receiver operating characteristic (ROC) curve of 0.77 (95%CI 0.64 - 0.89).^(19)^ Other authors also reported a significant association of the SOFA score with COVID-19 mortality.^(20)^

In this cohort, higher driving pressure (>14cmH_2_O) immediately after initiation of MV was associated with higher mortality, reinforcing that lower distension pressures imply a lower incidence of secondary injury induced by MV. The degree of pulmonary impairment in patients with ARDS heterogeneously reduces the useful lung area for ventilation, and thus, it is suggested that Vt should be adjusted accounting for these characteristics, ultimately reducing pulmonary strain. Consequently, lower distension pressures are generated, which has been shown to be associated with longer survival in this population.^(21)^ The driving pressure cutoff point established in this study was lower than that proposed using a previous cohort.^(21)^ In another cohort study conducted in Toronto, ^(22)^ there was an increase in the risk of death for each additional day in *driving pressure* ≥ 15cmH_2_O (RR 1.049 per day, 95%CI 1.023 - 1.076) or mechanical power ≥ 17J/minute (RR 1.069 per day, 95%CI 1.047 - 1, 092). Our results are comparable to those of another large cohort of patients with ARDS under MV with regard to their baseline characteristics and mortality rates.^(23)^

PaO_2_/FiO_2_ is used as a predictive factor to identify the presence of lung injury and to estimate the severity of hypoxemia. In this cohort, higher PaO_2_/FiO_2_ was associated with lower mortality. The mean PaO_2_/FiO_2_ of the participants in this study was low, revealing significant hypoxemia on admission. Severe hypoxemia has been one of the major obstacles of the disease, potentially presenting without changes in lung mechanics and responding differently to oxygen supplementation.^(24,25)^ Another study that evaluated the degree of hypoxemia among nonsurviving patients admitted to Wuhan Jin Yintan Hospital^(26)^ reported a lower PaO_2_/FiO_2_ ratio among the patients, which was associated with the mortality outcome.

Most patients in this cohort had low Crs, resulting in a higher probability of death at 28 days and 60 days (p < 0.001). Similar findings were also observed in previous studies.^(6,27,28,29)^ The impairment of lung mechanics can be explained by the evolution of COVID-19, which results in a hyperinflammatory state, and by the mechanisms that trigger patient-self-inflicted lung injuries (P-SILI), which are known to potentiate the lesion. ^(30)^ The change in Crs reflects the degree of heterogeneity and lung parenchyma and its relationship with the chest wall. This unfavorable mechanical characteristic and the increase in dynamic transpulmonary pressure are associated with ARDS severity.^(31)^

Our overall ICU mortality rate was 59.8% (95%CI 55.1 - 64.4). A systematic review^(32)^ that evaluated the characteristics and outcomes of hospitalizations for COVID-19 in Brazilian states reported that the mortality rate was 43% for patients admitted to the ICU and administered invasive M V, with higher mortality reported in public hospitals. The results herein were correlated with patients with obesity, low Crs, high driving pressure and high SOFA score at admission. In a Brazilian cohort of 574 patients, the mortality rate was 69.3% and was attributed to the number of comorbidities and disease severity.^(33)^ In a systematic review with meta-analysis, the combined mortality rate was 43% (95%CI 29 - 58), and the authors highlighted a strong association of invasive MV with acute kidney injury and ARDS in ICU outcomes.^(4)^ Another study reported a 35.7% improvement in mortality. ^(34)^ Some authors describe that improvements in outcomes over time are related to increased experience of professionals, to the establishment of admission and treatment criteria and to a reduction in demands on health systems, among other aspects.^(35)^

These results provide a snapshot and should be analyzed as data extracted from a historical cohort and with respect to the limitations that longitudinal studies of this nature have. Because this was an observational study, it was not possible to affect the control variables. In addition, the statistical analysis did not take into account where patients were treated, i.e., in public and private networks. Another limiting factor was the lack of access to information on patients who were immunized against COVID-19, which may have interfered with outcomes.

## CONCLUSION

Based on the data obtained in this study, patients with obesity, higher distension pressures and higher Sequential Organ Failure Assessment scores at the time of admission to initiating invasive mechanical ventilation had a lower probability of survival during follow-up. These variables, which were collected immediately after the initiation of invasive ventilatory support, resulted in worse outcomes and are independent risk factors associated with mortality in this population. These results may support not only treatment but also a better understanding of the prognosis of patients with acute respiratory distress syndrome.
